# Copeptin in Acute Myocardial Infarction: Is There a Role in the Era of High-Sensitivity Troponins?

**DOI:** 10.3390/jcdd12040144

**Published:** 2025-04-09

**Authors:** Sofia Bezati, Ioannis Ventoulis, Vasiliki Bistola, Christos Verras, Dionysis Matsiras, Effie Polyzogopoulou, John Parissis

**Affiliations:** 1 Department of Emergency Medicine, Attikon University Hospital, National and Kapodistrian University of Athens, 12462 Athens, Greece; sofiabezati@gmail.com (S.B.); christos.verras@gmail.com (C.V.); mats.dionysis@gmail.com (D.M.); iparisis@med.uoa.gr (J.P.); 2 Department of Occupational Therapy, University of Western Macedonia, 50200 Ptolemaida, Greece; iventoulis@uowm.gr; 3 Second Department of Cardiology, Attikon University Hospital, National and Kapodistrian University of Athens, 12462 Athens, Greece; vasobistola@yahoo.com

**Keywords:** copeptin, high-sensitivity troponin, acute myocardial infarction, biomarkers, vasopressin, dual marker strategy, rule-out, diagnostic tool

## Abstract

The quest for prompt and effective diagnosis of acute myocardial infarction (AMI) has been in the spotlight for decades. Ongoing research focuses on refined biomarker strategies for the early identification and disposition of patients with symptoms suggestive of AMI. Copeptin, a surrogate of the hormone arginine vasopressin, has emerged as a novel biomarker that could potentially aid in the diagnostic approach of patients with chest pain presenting to the emergency department. Observational studies have demonstrated that copeptin is upregulated in patients with AMI, although the exact pathophysiological mechanisms implicated in its release during myocardial ischemia remain unclear. Following these observations, copeptin was proposed as an adjunct to troponin in an effort to augment the diagnostic accuracy of conventional troponin assays. However, after the introduction of high-sensitivity troponin assays, the diagnostic utility of copeptin has been debated. This narrative review aims to elucidate plausible pathophysiological mechanisms involved in copeptin release during myocardial ischemia and to summarize the most recent evidence regarding its diagnostic potential in combination with high-sensitivity troponin assays.

## 1. Introduction

Ischemic heart disease continues to constitute a major healthcare challenge due to its high morbidity and mortality rates. Even nowadays, it is estimated to be the leading cause of death, accounting for 13% of global mortality [1]. In 2019, across Europe, cardiovascular disease was responsible for 11% and 15% of premature deaths in women and men, respectively, while there were 5.8 million new cases diagnosed with ischemic heart disease [2]. One of the most detrimental manifestations of ischemic heart disease is acute coronary syndrome (ACS), which necessitates prompt recognition and management. Since the introduction of the concept “time is muscle” [3], which emphasizes the fact that the duration of ischemia is directly related to the extent of myocardial injury, advancements in coronary reperfusion therapy have reduced mortality, especially in patients with ST segment elevation myocardial infarction (STEMI) [4,5]. However, non-ST segment elevation myocardial infarction (NSTEMI) is a field that requires further improvement [6] because epidemiological studies report conflicting data about NSTEMI mortality [7,8,9], essentially reflecting variations in the provision of care [10]. Caveats in the management of patients with NSTEMI include reduced adherence to NSTEMI guidelines [11], late patient presentation [12,13], and delayed delivery of appropriate therapy [14,15].

The emergency department (ED) holds a strategic role in the prompt evaluation of patients with undifferentiated chest pain, which accounts for approximately 10% of ED visits [16,17,18]. Among patients with chest pain, only a small proportion (around 10%) will eventually be diagnosed with NSTEMI [19,20]. However, their identification may be particularly challenging, considering that system-related obstacles, such as overcrowding, diagnostic ambiguities and risk of clinical misdiagnosis [15], may hinder the implementation of an appropriate approach. In order to overcome such difficulties, ongoing research has focused on risk stratification tools and biomarkers to reliably minimize the crucial time interval between patient presentation and definite diagnosis [21].

Cardiac troponin is a biomarker of myocardial injury typically associated with the diagnosis of ACS. According to the fourth universal definition of myocardial infarction, “detection of an elevated cardiac troponin value above the 99th percentile of the upper reference limit (URL) is defined as myocardial injury. In case the kinetics of cardiac troponin follow a rise and/or fall, the injury is considered acute” [22]. Consequently, diagnostic algorithms, which incorporate changes in troponin levels, have become the cornerstone in the diagnostic approach of patients with symptoms suggestive of ACS. Conventional troponin measurement is characterized by the “troponin-blind period”, defined as the time interval required to elapse between serial troponin measurements in order to detect abnormal elevations of troponin levels in case of acute myocardial infarction (AMI) [23]. To this end, diagnostic protocols initially required a time interval of 6 h between serial measurements of troponin. The introduction of high-sensitivity assays has resulted in the shortening of this time interval and in the earlier detection of even small AMIs [21] through the implementation of the shorter diagnostic protocols (0/1 h and 0/2 h) recommended by the 2020 guidelines of the European Society of Cardiology (ESC) [24].

However, gaps, uncertainties and limitations related to the application of troponin protocols have allowed the emergence of alternative biomarkers of myocardial injury and endogenous stress, which could serve as potential diagnostic tools in patients with ACS [25]. Copeptin is a non-specific biomarker reflecting endogenous stress, which has been proposed as an adjunct tool in the diagnostic workup of patients with chest pain [26]. The aim of this narrative review is to first summarize basic concepts regarding AVP and copeptin physiology, then propose potential pathophysiological mechanisms implicated in AVP/copeptin release in patients with AMI, and eventually provide most recent evidence on the diagnostic value of copeptin in combination with troponin, while discussing whether the use of copeptin confers any added benefit for the diagnosis of ACS in the era of high-sensitivity cardiac troponin assays.

## 2. Physiology

Copeptin was first isolated in 1972 by Holwerda et al. who reported the presence of a new neuropeptide extracted from the posterior pituitary gland of pigs, along with arginine vasopressin (AVP) and oxytocin [27]. Copeptin is a 39 amino acid-long C-terminal glycosylated peptide, which contains a leucine-rich core region. It is produced in equimolar proportions with AVP and neurophysin II upon cleavage of provasopressin (proAVP), the prohormone of vasopressin [28,29,30]. ProAVP is derived from a precursor peptide called preprovasopressin, after the removal of a signal peptide and the subsequent addition of a carbohydrate chain. These events take place in the hypothalamus [29,31]. ProAVP synthesis is triggered by hemodynamic or osmotic plasma alterations, as well as by stress-related signals, through two distinct mechanisms [29,32]. According to the first mechanism, proAVP is produced in the magnocellular neurons residing in the supraoptic nucleus (SON) and the paraventricular nucleus (PVN) of the hypothalamus. It is then folded and packed in neurosecretory vesicles and transported through the axons of the hypothalamus-pituitary tract down to the neurohypophysis. During this axonal transport, it is further processed and cleaved into its final peptide products, namely AVP, neurophysin II and copeptin, which are stored in neurosecretory granules in the neurohypophysis [31,33]. Subsequently, their release in the circulation is mainly driven by increased plasma osmolarity and by changes in extracellular fluid volume [30]. Alternatively, proAVP may also be produced and processed in the parvocellular neurons of the hypothalamus and then secreted to the pituitary portal system, whereupon AVP acts on the endocrine cells of the adenohypophysis [29,33,34]. Interestingly, AVP is produced in the same hypothalamic areas as corticotropin-releasing hormone (CRH) [33], which may account for the fact that both of these hormones demonstrate a synergistic stimulatory effect on the release of adrenocorticotropic hormone (ACTH) from the anterior pituitary gland and eventually the secretion of cortisol from the adrenal glands [34,35]. This second mechanism explains why AVP is considered to be involved in the endocrine stress response induced by non-specific stressful conditions such as pain, tissue damage and inflammation [36].

AVP exerts its biological effects by binding to several G-protein-coupled receptor subtypes, namely the vascular V1 (or V1a), the renal V2, the pituitary V3 (or V1b) and the oxytocin (OT) receptors [37]. V1 receptors are abundant in vascular smooth muscle cells and induce vasoconstriction upon AVP activation. Their presence has also been reported in other cells and tissues, such as the liver, kidney, central nervous system, superior cervical ganglion and platelets, but their effects on these tissues are less well-characterized [38]. V2 receptors are found in abundance on the basolateral cell membrane of renal collecting tubules, where they mediate the antidiuretic effects of AVP. V3 receptors are mainly located in the anterior pituitary gland and are involved in the release of ACTH [38,39]. V1 and V3 receptors are also found in the adrenal cortex (only V1) and medulla (both V1 and V3), mediating steroid and catecholamine release upon stimulation by locally secreted AVP [40]. OT receptors have a similar affinity for oxytocin and AVP and may be regarded as non-selective AVP receptors. They are present in myometrial and mammary myoepithelial cells, lactotroph cells, as well as in the brain and the kidney. They are also densely expressed in the vascular endothelium, facilitating nitric oxide (NO)-dependent vasodilatation [37,38]. Additionally, AVP has also been shown to act on a new class of endothelial purinergic receptors (P2), thereby affecting both cardiac contractility and vascular reactivity in a variable manner [41,42,43].

Although the effects of AVP have been thoroughly described thus far, the role of copeptin is still not well-understood. Most scientific evidence points towards the fact that copeptin lacks direct physiologic effects and rather has an adjunctive role in optimizing efficient folding and promoting the correct structural formation of proAVP during its proteolytic maturation [29,33,44]. Nowadays, it is considered a surrogate marker of the activation of the vasopressin system in various clinical conditions, including polyuria-polydipsia syndromes, hyponatremia, heart disease (AMI and heart failure), shock states, polycystic kidney disease and diabetes mellitus [29].

## 3. Proposed Pathophysiological Mechanisms Leading to AVP/Copeptin Release in Myocardial Ischemia

The exact pathophysiological mechanisms responsible for the release of AVP (or its surrogate copeptin) in patients with myocardial ischemia have not been fully elucidated. Interestingly, in an experimental rat model of AMI, it was demonstrated that magnocellular AVP- and oxytocin-producing neurons residing in the hypothalamic SON and PVN were activated immediately after the induction of AMI [45]. Additionally, in a human study, which included two groups of patients with or without AMI who underwent coronary angiography, it was found that patients with AMI had significantly higher levels of copeptin compared with the group of non-AMI patients. By measuring the concentration of copeptin simultaneously in both the aortic bulb and the coronary venous sinus and calculating the transcoronary gradient of copeptin levels, the same study reported that copeptin was not locally released from the heart into the circulation. Thus, the researchers concluded that the origin of elevated copeptin was non-cardiac and could, therefore, be attributed to a centrally mediated mechanism originating from the central nervous system (CNS) [46].

Myocardial ischemia is caused by either reduced coronary perfusion or a mismatch between oxygen demand and supply. Regardless of the underlying mechanism, the net result of myocardial ischemia is myocardial tissue hypoxia, shift to anaerobic metabolism, lactic acidosis [47] and inflammation following myocardial injury [48]. On this account, the most plausible explanation for the secretion of AVP in the setting of cardiac ischemia could be a generic endocrine stress response triggered by the aforementioned state of altered cardiac homeostasis, eventually resulting in the non-specific elevation of AVP [33,49]. This theory has been corroborated by the findings of Katan et al., who conducted a clinical study including healthy subjects and patients with different degrees of critical illness. The authors reported that copeptin correlated with the individual stress level incurred by the underlying disease. Indeed, there was a progressive increase in copeptin levels according to disease severity, while a correlation between copeptin and cortisol levels was also evident. Even more so, copeptin was able to discriminate healthy subjects from patients with moderate stress levels, whereas cortisol failed to do so [50]. In addition, the well-known interaction of AVP with the hypothalamic–pituitary–adrenal (HPA) axis during stress [51] further supports the notion that AVP is upregulated in myocardial ischemia owing to an exaggerated neuroendocrine response to ischemia-induced stress [33]. Within this framework, copeptin, the surrogate marker of AVP, actually reflects endogenous stress levels [34].

In detail, ischemia-induced stress causes the release of both CRH and AVP from the parvocellular neurons of the hypothalamus. AVP is then transported through the pituitary portal system down to the adenohypophysis and stimulates the corticotroph cells of the anterior pituitary through V3 receptors to secrete ACTH, further potentiating the CRH-induced ACTH release. In turn, ACTH exerts its effects on the adrenal glands for the release of cortisol [51,52]. Apart from its action on the CNS, AVP may also act directly on the adrenal glands and evoke the secretion of cortisol through V1 receptors located in the adrenal cortex, as well as the secretion of catecholamines through V1 and V3 receptors residing in the adrenal medulla [40]. By doing so, AVP may additionally exert its action in an autocrine and paracrine manner, given that it has been shown that AVP is secreted not only by the CNS but also by the chromaffin cells in the adrenal medulla [52].

The hypothesis of the activation of the HPA axis in response to ischemia-induced stress is supported by the findings of an experimental study in an ovine model of myocardial infarction. Following microembolization of the coronary arteries, it was observed that the plasma levels of AVP, ACTH and cortisol were elevated within 2 h, with the peak responses occurring 40 min after embolization. Plasma concentrations of ACTH and cortisol had decreased by 6 h, whereas AVP levels remained elevated for more than 12 h [53]. In addition, an increase in plasma levels of AVP, CRH, ACTH and cortisol has also been demonstrated in humans following an AΜΙ [54].

In a similar vein, the increase of AVP during AMI could be attributed to the inflammatory cascade provoked by the underlying myocardial ischemia. Indeed, following AMI, an inflammatory response ensues due to myocardial injury, which results in a cascade of humoral and cell-mediated events, including complement activation, release of chemotactic factors, generation of free radicals and reactive oxygen species, release of cytokines, recruitment of inflammatory cells and infiltration of the myocardium [55]. This inflammatory cascade leads to the upregulation of proinflammatory cytokines, which have been shown to mount a neuroendocrine response with activation of both the sympathetic nervous system and the HPA axis as part of the endocrine stress response. Stimulation of the HPA axis leads to the augmented release of AVP and CRH, which synergistically trigger the release of ACTH and, subsequently, cortisol in an attempt to suppress inflammation [56]. Additionally, AVP has been shown to serve as a neuroendocrine-immune mediator, exerting various immunomodulatory actions, some of which entail anti-inflammatory effects [52,57]. In this regard, the secretion of AVP could represent a possible protective mechanism in the early phase of AMI, activated in order to prevent or dampen a potentially detrimental flare of an excessive inflammatory response [58].

Another putative pathophysiological mechanism that might explain the rise of AVP and copeptin in myocardial ischemia could be based on the rationale that AVP has a modulatory role on the coronary vascular tone, although experimental studies have reported contradictory results [59]. Animal studies have demonstrated that AVP can induce either vasodilatation [60] or vasoconstriction [61] of the coronary arteries. Coronary vasoconstriction is believed to be mediated mainly through V1 receptors, whereas vasodilation is endothelium-dependent and seems to be mediated mainly by OT receptors (through activation of the endothelial NO system), as well as by P2 purinergic receptors at low AVP doses (through ATP as an intermediary) [43,59]. However, it has also been reported that stimulation of the P2 purinergic receptors in the heart by AVP can augment perfusion pressure by causing vasoconstriction of the coronary arteries [41]. Given the controversial effects of P2 purinergic receptors, AVP seems to elicit coronary vasodilation predominantly through endothelial OT receptors on the grounds that it has a close affinity for these receptors, which mediate NO-dependent vasodilation [33,62]. Therefore, the net effect of AVP on coronary arteries could be the result of the interaction among V1, P2 and OT receptors.

On this account, it has been reported that the vasoconstrictive effect of AVP prevails in normoxic states, whereas the vasodilatory effect is observed in hypoxic states [59,63]. During AMI, diminished coronary blood flow leads to reduced oxygen supply to the myocardium and subsequent tissue hypoxia [64]. Under these hypoxic conditions, the release of AVP may be a protective mechanism, which could be activated as a means to restore coronary blood flow and oxygen tension in the stressed myocardium by preferentially causing the appropriate degree of coronary vasodilation. There is also a possibility that AVP may exert bimodal actions by causing some degree of vasoconstriction in certain beds of the coronary vasculature and vasodilation in others as a mechanism of redistributing blood flow to the oxygen-deprived areas. By doing so, AVP may selectively constrict flow to non-ischemic myocardial areas and redirect flow to ischemic myocardial regions [63]. In this regard, AVP upregulation in myocardial ischemia may represent an intricate mechanism that results in the fine-tuning of the coronary vascular tone by exploiting both the vasoconstrictive and vasodilatory effects of AVP. It is plausible that AVP elaborately modulates the vascular tone to such an extent that it conserves adequate perfusion to ischemic areas on the one hand while at the same time avoiding excessive vasodilation that would impair post-obstruction perfusion pressure and elicit a “coronary-steal” phenomenon through reduction of blood flow to the ischemic areas [63]. To this end, the bimodal actions of AVP might actually be elicited simultaneously and function in a synergistic manner in order to elegantly retain an appropriate balance between coronary vasoconstriction and vasodilation [59]. This proposed theory of an AVP protective mechanism to restore coronary blood flow during myocardial ischemia, either by inducing selective coronary vasodilation or by appropriately redistributing blood flow while maintaining coronary perfusion pressure through both vasoconstrictive and vasodilatory actions, definitely deserves further clarification.

Apart from the postulated mechanism of the AVP-mediated protective effect on coronary blood flow, one might also speculate that cardiac ischemia may induce AVP release due to impaired cardiac contractility and reduced cardiac output, which in turn may result in a drop in blood pressure, stimulation of arterial baroreceptors and activation of the autonomous nervous system (ANS), thereby modulating AVP secretion. Cardiopulmonary baroreceptors may also be directly injured due to myocardial ischemia and elicit an inappropriate ANS response [19,33].

An alternative pathophysiological mechanism that could justify the upregulation of AVP in myocardial ischemia may be related to the fact that AVP has been implicated in the processing of pain sensation and transmission. Modulation of cardiac ischemic pain is a complex phenomenon involving the activation of the ANS and the release of AVP [65]. It is believed that AVP may be directly secreted from the hypothalamus in response to pain because nociceptive signals may ascend to the hypothalamic SON via the caudal ventrolateral medulla and the noradrenergic A1 region and enhance AVP release [66]. In addition, AVP-producing neurons in the SON, PVN and suprachiasmatic nucleus (SCN) are in a constant interplay with neurons residing in other areas of the CNS, which are involved in pain regulation, such as the amygdala, nucleus tractus solitarius (NTS), caudate nucleus, periaqueductal grey (PAG) and cingulate gyrus [65]. Accumulating evidence indicates that AVP possesses analgesic properties and plays a pivotal role in pain modulation [67]. There have been reports from animal studies that upregulation of AVP conveyed an analgesic effect in rats after induction of painful stimuli. This effect was abolished by V1 receptor antagonism, thus implying that the antinociceptive effect of AVP was mediated through V1 receptors [68,69]. It has also been reported that centrally released AVP increases the pain threshold [65] through interaction with neurons involved in pain modulation and located in central brain areas, such as the amygdala [70], PAG [71] and caudate nucleus [72]. PET studies on patients with myocardial ischemia show that brain areas involved in pain perception include the hypothalamus, the thalami, the PAG, the prefrontal cortex and the left inferior antero-caudal cingulate cortex [73]. As cardiac ischemic pain is controlled by brain areas located in the vicinity of the AVP production site, it is plausible that stimulation of AVP and subsequent release into the plasma of patients with AMI is triggered by nociceptive stimuli to exert its analgesic effect or even affect the tone of the coronary vasculature.

**Figure 1:**
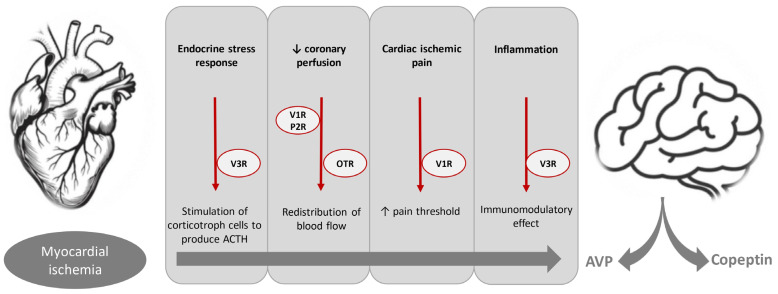
Postulated pathophysiological mechanisms implicated in AVP/copeptin release during myocardial ischemia. ↓: decrease; ↑: increase.

Figure 1 provides a schematic illustration of the proposed pathophysiological mechanisms implicated in AVP/copeptin release in patients with myocardial ischemia.

## 4. The Assay

Former experimental studies seeking to explore the association between AVP and AMI reported an increase in AVP levels during the initial phase of AMI; however, the lack of an appropriate assay to measure AVP levels hindered further investigation [33]. In 2006, a novel sandwich immunoluminometric assay (LIA) for the quantification of copeptin was introduced, which was able to provide results within 3 h. The assay had a lower detection limit (LoD) of 1.7 pmol/L and a 20% functional assay sensitivity of 2.25 pmol/L. Namely, the interlaboratory coefficient of variation (CV) was <20% for values >2.25 pmol/L. In healthy individuals, median copeptin levels were 4.2 pmol/L [interquartile range (IQR) 1–13.8 pmol/L; 95% confidence interval (CI) 4.0–4.4 pmol/L]. For better clinical interpretation of the results, it should be mentioned that the 99th percentile of the healthy population was 13.5 pmol/L, the 97.5th percentile was 11.25 pmol/L, and the 2.5th percentile was 1.7 pmol/L [74]. Later, after technical modifications of the assay, its 20% functional sensitivity improved to <1 pmol/L, while the LoD was reported to have reached a value of 0.4 pmol/L [75].

Later on, technical advancements in copeptin assays led to the development of a fully automated immunofluorescent assay for the quantitative measurement of copeptin on the Kryptor platform through the use of Time-Resolved Amplified Cryptate Emission Technology (TRACE) [76]. This assay was further refined, and currently, an ultra-sensitive assay for measuring copeptin on the automated Kryptor Compact Plus system (Thermofisher Scientific, Clichy, France) is commercially available. The latter assay is able to measure copeptin within 15 min, with a LoD of 1.9 pmol/L (0.9 pmol/L as claimed by the manufacturer [77]) and a 10% functional sensitivity of 3 pmol/L [78]. These technical characteristics render it superior to the conventional LIA assay in terms of analytical performance. It is worth mentioning that the LIA and the Kryptor assays provide comparable results on copeptin plasma concentrations [79].

## 5. Copeptin and Acute Myocardial Infarction

As soon as measurement of copeptin became feasible [74], Khan et al. made a novel observation that copeptin rises early in patients with AMI and may offer additional prognostic information. Indeed, copeptin levels were significantly higher in patients with AMI compared with healthy individuals [7.0 pmol/L (0.3–441) vs. 3.8 pmol/L (0.44–44.3), respectively, *p* < 0.0005]. The study group performed serial measurements of copeptin and reported that copeptin peaks on the first day after AMI and then reaches a plateau by days 3 to 5. Moreover, higher copeptin levels were significantly correlated with the diagnosis of STEMI, the development of heart failure and death [80]. Additionally, copeptin levels have been found to be positively associated with the myocardial infarct size and the process of adverse remodeling in patients with STEMI [81], as well as with the angiographic severity of coronary artery disease in patients with unstable angina [82].

### 5.1. Evidence Establishing the Combined Use of Copeptin with Conventional Troponin

The landmark study, which triggered further research regarding the diagnostic utility of copeptin in patients with AMI, was conducted in 2009 by Reichlin et al. who investigated the added value of copeptin on top of conventional troponin T for the rapid rule-out of patients with chest pain. Patients with AMI had higher levels of copeptin compared with patients with other diagnoses. Amongst patients with AMI, STEMI patients had significantly higher copeptin values in comparison with NSTEMI patients [45.5 (IQR 21–123) vs. 11.7 (IQR 6.2–50.8) pmol/L]. The combination of copeptin with conventional troponin T resulted in a significantly enhanced diagnostic accuracy compared with conventional troponin T alone, as evidenced by an area under the curve (AUC) of 0.97 (95% CI 0.95–0.98) vs. 0.86 (95% CI 0.80–0.92) respectively. A copeptin value <14 pmol/L combined with a troponin T value ≤0.01 μg/L could reliably exclude AMI with a sensitivity of 98.8% and a negative predictive value (NPV) of 99.7%. Of note, copeptin levels were higher during the first 4 h after symptom onset and decreased afterward, whereas troponin levels followed an inverse pattern. It is of great clinical importance that patients with AMI presenting early to the ED already had increased levels of copeptin, while troponin levels were still undetected [75].

This latter observation was actually an important milestone that laid the foundations for introducing the innovative concept that NSTEMI could be ruled out upon ED presentation by adopting a dual marker strategy (DMS) without the need for serial troponin sampling. The rationale for incorporating copeptin in the diagnostic approach of patients with symptoms suggestive of AMI lies in its potential use as an early marker for rapid rule-out. This unique feature of copeptin is attributed to its kinetics, given that it is elevated in the first hour after AMI and subsequently reaches normal values in approximately 20 h [19,83]. This has also been documented in the prehospital setting, where copeptin levels peaked very early, during the first medical contact of patients with AMI in the ambulance, and decreased rapidly thereafter within the first hours. This resulted in very high diagnostic performance (AUC 0.963 and NPV 100%) of copeptin as a stand-alone marker in patients who were early presenters, whereas in late presenters, the NPV of copeptin was only 50% [84].

In line with the above, Keller et al. studied the role of copeptin as a diagnostic marker in 1386 patients with suspected ACS and found that the combination of copeptin and conventional troponin T exhibited better diagnostic performance for detection of AMI (AUC 0.93) compared with conventional troponin T alone (AUC 0.84). The diagnostic power of the combined use of copeptin and conventional troponin T was even more pronounced in patients presenting with chest pain onset of less than 3 h because it resulted in an AUC of 0.90 compared with an AUC of 0.77 when using conventional troponin T alone. In the same study, the investigators examined the performance of several potential diagnostic cut-off values for copeptin, which were chosen based on the Gutenberg Heart study comprising 5000 individuals. The applied cut-offs for copeptin were 18.9, 13 and 9.8 pmol/L, corresponding to the 99th, 97.5th and 95th percentiles, respectively. They concluded that a copeptin value of 9.8 pmol/L, which corresponded to the 95th percentile, had the highest sensitivity (85.1%) and NPV (92.4%) when applying the DMS (copeptin plus conventional troponin T) in patients presenting with chest pain onset of <3 h. Considering the high NPV of the combined use of copeptin and troponin T, which was found to be virtually independent of chest pain onset, the implementation of a combined strategy based on both copeptin and conventional troponin T could cover the diagnostic uncertainty resulting from the “troponin-blind” period [85]. Apart from conventional troponin T, copeptin was also studied in combination with conventional troponin I in a cohort of 1967 patients presenting with chest pain, and it was demonstrated that the combined use of copeptin and conventional troponin I also exhibits high diagnostic potential in terms of ruling out AMI, with an NPV of >99% [19].

Additionally, in a multicenter randomized controlled clinical trial investigating the safety of early discharge using a DMS, results were also encouraging with regard to the use of copeptin in combination with troponin (conventional or high-sensitivity troponin). They showed that a DMS based on a single combined testing of troponin and copeptin is non-inferior to the standard strategy of serial troponin testing in terms of ruling out AMI in patients with low to intermediate risk for ACS. Indeed, the proportion of patients with major adverse cardiac events did not differ between the two groups, while the DMS resulted in a shorter length of stay in the ED and led to a greater number of discharges directly from the ED [86].

Furthermore, a prospective multicenter study investigated whether the diagnostic and prognostic performance of copeptin in combination with conventional troponin T or high-sensitivity troponin T (hs-cTnT) differed in men and women. Among various diagnostic combinations (conventional troponin T alone; hs-cTnT alone; copeptin alone; copeptin with conventional troponin T; copeptin with hs-cTnT), it was found that the combination of copeptin with either conventional troponin T or hs-cTnT had the best diagnostic accuracy (AUC 0.96) regardless of sex [87].

Moreover, in a systematic review and meta-analysis examining the incremental value of copeptin for the exclusion of AMI in 9244 patients enrolled in 14 studies between 2010–2013, the addition of copeptin to troponin resulted in enhanced sensitivity and NPV for AMI compared with troponin alone, regardless of the troponin assay used (be it conventional or high-sensitivity troponin assay). However, when assessing the pooled area under the receiver operating characteristic (ROC) curves, only the combination of copeptin with conventional troponin (but not the combination of copeptin with high-sensitivity troponin) yielded better diagnostic accuracy for AMI compared with conventional troponin alone (0.91 vs. 0.86, respectively, *p* = 0.02, I^2^ = 83%). As expected, it was also observed that patients with AMI had significantly greater median values of copeptin (22.8 pmol/L) compared with patients without AMI (8.3 pmol/L). Interestingly, there were no differences in the sensitivity, specificity, NPV, AUC, positive and negative likelihood ratio for the cut-off points adopted for copeptin (<14 or <10 pmol/L) across the different studies, suggesting that either cut-off could be used without influencing the diagnostic accuracy of the test [88].

Likewise, another systematic review and meta-analysis, which included 15 studies with a total of 8740 patients presenting with symptoms suggestive of AMI, reported that the combination of copeptin with troponin (both conventional and high-sensitivity troponin) led to significant improvement of the baseline troponin sensitivity, albeit at the expense of lower specificity. Even when analyzing the subgroup of studies using exclusively hs-cTnT, it was still observed that the addition of copeptin offered significant incremental diagnostic accuracy for AMI, resulting in a significant increase in the baseline sensitivity of hs-cTnT [89].

Based on certain studies mentioned above [19,75,85,86,87,88,89], which documented the incremental value of copeptin on the diagnostic accuracy of conventional troponins T and I, the DMS was eventually incorporated in the ESC guidelines for the assessment of patients with NSTEMI released in 2015 [90]. Along the same lines, the latest ESC guidelines in 2020 [24] recommend the measurement of copeptin only in institutions where high-sensitivity troponin assays are lacking. Hence, they recommend against the use of copeptin in clinical settings where high-sensitivity troponin assays are available due to inconsistent data regarding its additional diagnostic utility.

Table 1 summarizes the main characteristics of the studies that have provided evidence for the combined use of copeptin with conventional troponin.

### 5.2. Copeptin in Combination with High-Sensitivity Troponin

Evidence on the incremental value of copeptin, when used in combination with high-sensitivity troponin assays, has been rather contradictory, with some studies supporting its moderate diagnostic contribution and others reporting the absence of any diagnostic benefit. The first study to report a beneficial effect of using copeptin in combination with high-sensitivity troponin dates back to 2011 when Meune et al. conducted a pilot study in 57 patients with chest pain with the aim to investigate the possible added value of copeptin on top of hs-cTnT for the detection of ACS. Combined measurement of both biomarkers on admission resulted in a slightly better, yet statistically not significant, diagnostic accuracy in comparison to baseline measurement of hs-cTnT alone, as suggested by an increase in the AUC value from 0.90 to 0.94. The DMS demonstrated equal diagnostic performance when compared to serial measurements of hs-cTnT [91].

#### 5.2.1. Evidence Suggesting That DMS Is Superior or Equal to High-Sensitivity Troponin Protocols

Following the aforementioned observation, further studies sought to elucidate this observation in larger-scale clinical trials. In 2013, Sebbane et al. conducted a prospective single-center study in 194 patients presenting to the ED with chest pain and concluded that a DMS using a copeptin cut-off value of 13.11 pmol/L in combination with hs-cTnT > 14 ng/L yielded better diagnostic accuracy in comparison to a single measurement of hs-cTnT at baseline. The estimated AUC for AMI diagnosis increased significantly from 0.89 to 0.93, while sensitivity and NPV for NSTEMI diagnosis also improved. Sensitivity increased from 76% to 96% and NPV from 95.3% to 98.9% for hs-cTnT alone vs. DMS, respectively, thus suggesting that the DMS could potentially be used for early and safe rule-out of NSTEMI [78].

The diagnostic performance of copeptin was also tested in patients with certain comorbidities. In a prospective study including 433 patients with chest pain and a history of coronary artery disease, the addition of copeptin to hs-cTnT showed a trend towards enhancing diagnostic work-up because it yielded an AUC of 0.94 compared with an AUC of 0.92 for hs-cTnT alone, albeit failing to reach statistical significance. Nevertheless, by using 9 pmol/L as a cut-off for copeptin, the combination of the two biomarkers improved both the sensitivity of baseline hs-cTnT (from 93.6 to 98.7) and NPV (from 97.7 to 99.3) [92]. Likewise, in a prospective multicenter study that enrolled 379 diabetic patients with suspected AMI, the addition of copeptin (cut-off 9 pmol/L) to hs-cTnT did not provide any further diagnostic benefit, given that the combined use of copeptin and hs-cTnT exhibited comparable diagnostic performance to hs-cTnT alone, yielding identical AUC values for the detection of AMI. On the contrary, adding copeptin to conventional troponin T resulted in superior diagnostic accuracy compared with conventional troponin alone [93].

Taking into consideration that copeptin peaks early after chest pain onset, its diagnostic potential was investigated in the prehospital setting in 962 patients with symptoms suggestive of AMI. The sensitivity and NPV of copeptin alone were higher in patients with symptom onset <1 h and decreased thereafter. Moreover, the combination of copeptin (<9.8 pmol/L) and hs-cTnT (<14 ng/L) demonstrated a significantly better AUC (0.85) than hs-cTnT alone (0.81) and led to a safe rule-out of 45% of patients with a sensitivity of 96% and an NPV of 98% [94].

An international multicenter prospective study, which included 1929 patients with chest pain, explored the incremental diagnostic benefit of combining copeptin with six different troponin I assays [three conventional and three high-sensitivity cardiac troponin I (hs-cTnI) assays] as compared with the use of the corresponding troponin I alone. The combination of the two biomarkers yielded better diagnostic accuracy, as indicated by AUC values, only when two of the conventional troponin I assays were used in combination with copeptin, whereas no benefit of the DMS (in terms of AUC) was observed for any of the hs-cTnI assays. However, the results were different when the authors performed an integrated discrimination improvement (IDI) statistical analysis, which is regarded as a less subjective test than the ROC curve analysis, owing to the fact that it is not dependent on the existence of meaningful risk categories. By using this method, it was observed that IDI increased significantly for all troponin I assays when combined with copeptin. Sensitivity and NPV also increased when copeptin was added to all hs-cTnI assays. The study group concluded that copeptin conferred a slight, but still significant, added benefit for rapid rule-out of NSTEMI when combined with hs-cTnI assays [95].

With the advent of high-sensitivity troponin assays, new diagnostic algorithms emerged, further challenging the diagnostic utility of copeptin. Accordingly, a prospective observational study, which included 270 patients presenting to the ED with chest pain, examined the diagnostic accuracy of a DMS that combined copeptin and hs-cTnI vs. the 0/2 h algorithm based on the measurement of hs-cTnI. The results showed equal sensitivity (100%) and NPV (100%) for both strategies, hence indicating that the DMS was non-inferior to the 0/2 h algorithm [96]. A sub-analysis of the high sensitivity cardiac Troponin T assay for RAPID rule-out of Acute Myocardial Infarction study (TRAPID-AMI study) assessed the diagnostic performance of the DMS, namely copeptin combined with hs-cTnT (≤14 ng/L) upon presentation, by applying different cut-off levels for copeptin (<10 pmol/L, <14 pmol/L and <20 pmol/L) and by comparing DMS with several diagnostic protocols: (1) baseline hs-cTnT ≤14 ng/L (99th percentile) alone; (2) baseline hs-cTnT <3 ng/L, which is the value below the limit of blank (LoB); (3) baseline hs-cTnT < 5 ng/L, which is the value below the limit of detection (LoD); (4) hs-cTnT < 12 ng/L and absolute delta change <3 ng/L at 1 h (1 h algorithm); and (5) hs-cTnT < 5 ng/L when chest pain onset is >3 h, or else hs-cTnT <12 ng/L and absolute delta change <3 ng/L at 1 h [97], as per the 2015 ESC guidelines for NSTEMI [90]. The overall diagnostic performance for AMI did not differ between the strategy combining copeptin with hs-cTnT and the strategy using only hs-cTnT, given that DMS upon presentation had an estimated AUC of 0.93, which was similar to the AUC of hs-cTnT alone (0.92) [97]. The best sensitivity (94.8%) and NPV (98.3%) were achieved at a copeptin threshold of 10 pmol/L, which resulted in the safe exclusion of AMI in 50.9% of patients. Notwithstanding, it was observed that sensitivities and NPVs of the different copeptin cut-offs were not significantly different, with sensitivities ranging from 93.5–94.8% and NPVs ranging from 98.1–98.3%. This suggests that a copeptin cut-off of 20 pmol/L could also be used, yielding a rule-out rate of 62.3%. When comparing the DMS with the other diagnostic algorithms, it was shown that the true rule-out rates of the other strategies were as follows: 68.8% for the strategy of using only baseline hs-cTnT ≤ 14 ng/L; 35% for the strategy of using baseline hs-cTnT < 3 ng/L; 45.3% for the strategy of using baseline hs-cTnT < 5 ng/L; 64.5% for the 1 h algorithm; and 43.1% for the 2015 ESC algorithm for NSTEMI. Regarding sensitivities and NPV of the other diagnostic strategies, a single measurement of hs-cTnT ≤ 14 ng/L yielded a sensitivity of 89% and an NPV of 97.4%, both of which increased to 94.8% and 98.3%, respectively, with the addition of copeptin (10 pmol/L). The NPVs of the remaining strategies were >99% and sensitivities ranged from 96.8% to 98.7%. The algorithm proposed by the 2015 ESC guidelines for NSTEMI was found to be the most sensitive, albeit with the limitations of necessitating two troponin measurements and being capable of ruling out a smaller percentage of patients (43.1% vs. 50.9% when implementing the DMS). Improvement of the diagnostic accuracy of the DMS could be achieved by adding a HEART [98] score ≤ 3 or a GRACE [99] score < 109 to the DMS during the initial assessment, yet at the expense of ruling out a smaller proportion of patients [97].

Furthermore, Giannitsis et al. accumulated data from five studies, resulting in a total of 10,329 patients with symptoms suggestive of AMI. The primary endpoint of the study was to compare sensitivities and NPVs, 30-day all-cause mortality and effectiveness among three different strategies: the DMS, using a copeptin value <10 or 14 pmol/L (depending on the assay used) together with a troponin (either conventional or high-sensitivity troponin) value <99th percentile; a strategy of measuring at baseline either conventional or high-sensitivity troponin alone based on a cut-off <99th percentile; and a single marker strategy based on a single very low high-sensitivity troponin value using a cut-off <LoD. The DMS (regardless of the troponin assay used) resulted in significantly higher NPVs and sensitivities for NSTEMI rule-out compared with the strategy of measuring high-sensitivity troponin alone (with a cut-off <99th percentile) at baseline. The DMS, which was based on the addition of copeptin to high-sensitivity troponin, yielded the highest NPV (99.4%) and sensitivity (96.4%), whereas the corresponding values for high-sensitivity troponin alone were 98.8% (NPV) and 90.2% (sensitivity). Regarding comparisons between the DMS and the single marker strategy using a very low high-sensitivity troponin value (<LoD), the two protocols yielded equal NPVs (99.4% vs. 99.6%, respectively). With respect to 30-day all-cause mortality, all three strategies showed excellent NPV (>99.75%). The indisputable advantage of the DMS in this study was its wider applicability in patients with suspected ACS, considering that it could effectively rule out 61.4% of the patients, as opposed to only 25.3% rule-out effectiveness of the single marker strategy based on very low high-sensitivity troponin. The authors also emphasized the fact that, compared with high-sensitivity troponin alone, the DMS demonstrated equal diagnostic performance in very early presenters, irrespective of age, sex or comorbidities like chronic kidney disease, diabetes mellitus and coronary artery disease [100,101].

Other studies focused on the safety and efficacy of the DMS in comparison to various rule-out troponin protocols. Following the randomized controlled trial by Möckel et al., who demonstrated the safety of implementing a DMS (based on the addition of copeptin to conventional or high-sensitivity troponin assays) in 902 low-to-moderate risk patients with suspected ACS [86], a larger prospective multicenter study, the ProCore registry, was conducted, which included 2294 patients from 18 European EDs [102]. This study examined the safety of applying a DMS in low-to-intermediate risk patients presenting with a broad range of symptoms suggestive of ACS. The DMS was based on combining copeptin (at a cut-off limit of 10 pmol/L) with a wide spectrum of cardiac troponin assays, namely conventional, point-of-care or high-sensitivity troponin assays, at a cut-off value <99th percentile, thus reflecting real-world daily practices. The authors found that the DMS could safely rule out 42.5% of the patients, similar to the 0/1 h algorithm. The all-cause mortality rate was minimal (0.1%) in patients eligible for early rule-out based on the DMS. Notably, the NPV of the DMS was 99.9% for the exclusion of NSTEMI [102]. Likewise, Ricci et al. performed a direct comparison between a DMS (copeptin < 10 pmol/L and hs-cTnI < 99th percentile) and a single marker strategy using hs-cTnI with a threshold of <5 ng/L. They studied the safety and effectiveness of both strategies for ruling out AMI in 1136 patients presenting with chest pain. The two protocols did not demonstrate any significant differences in their sensitivities and NPVs and exhibited similar diagnostic performance, which was comparable to the 0/1 h ESC algorithm. While the safety of the implementation of the two protocols was similar to the 0/1 h ESC algorithm, the DMS provided a slight, yet significant, benefit over the single marker strategy in terms of effectiveness in ruling out AMI. As a matter of fact, the DMS effectively ruled out 37.4% of the patients compared with the 32.9% rule-out rate achieved by the single marker strategy [103].

Table 2 summarizes the main features of the studies showing that DMS is superior or equal to high-sensitivity cardiac troponin protocols.

#### 5.2.2. Controversial Evidence or Evidence Suggesting That DMS Is Inferior to High-Sensitivity Troponin Protocols

In a study of 2000 patients complaining of symptoms suggestive of AMI, the addition of copeptin on top of hs-cTnT did not improve diagnostic accuracy for AMI because the DMS yielded an AUC of 0.86 compared with an AUC of 0.87 for hs-cTnT alone. By applying the DMS and using a cut-off value of 9 pmol/L for copeptin and 14 ng/L for hs-cTnT, only the NPV increased. The DMS yielded higher NPV and AUC in patients presenting >2 h from chest pain onset compared with those presenting early (<2 h), probably due to the enhanced diagnostic performance of hs-cTnT, which is time-dependent and increases over time and not due to the effect of copeptin per se. Therefore, this study provides inconclusive information regarding the diagnostic performance of the DMS as a rule-out strategy [105].

In addition, copeptin has also been examined in the context of an alternative rule-out strategy, which was based on serial measurements of hs-cTnT and copeptin at two different time points: upon ED presentation (baseline) and after one hour. The study included 1439 patients with chest pain (105 of whom presented within 2 h from chest pain onset). At baseline, the NPV for hs-cTnT alone was 97.1%, whereas the NPV for the combination of hs-cTnT and copeptin was 98.6%. A second measurement of copeptin at 1 h did not result in any significant improvement of the NPV for AMI, whereas a second measurement of hs-cTnT at 1 h did, in fact, increase the NPV to 99.6% [106]. Results regarding the utility of performing a second measurement of copeptin as an early rule-out strategy might actually be discouraging, but they are fully justified based on the kinetics of copeptin [83,84]. So, even if the design of this study was to propose an alternative rule-out strategy for AMI, the most important finding was the confirmation of the incremental value of copeptin when combined with hs-cTnT levels upon ED presentation in terms of yielding better NPV for AMI [106].

Boeddinghaus et al. conducted a subanalysis of the Advantageous Predictors of Acute Coronary Syndrome Evaluation (APACE) multicenter trial and focused exclusively on the group of patients presenting with suspected AMI and mild elevations of hs-cTnT (14–50 ng/L) and/or point of care hs-cTnI (26.2–75 ng/L). In this population, copeptin levels did not differ significantly between patients who were eventually diagnosed with AMI and those who subsequently received another diagnosis (median copeptin values 20.3 pmol/L vs. 13.3 pmol/L, respectively, *p* = 0.23). The diagnostic accuracy of hs-cTnI, copeptin or their combination was only modest, as evidenced by their AUC values (0.51, 0.58 and 0.52, respectively). On the other hand, the AUC corresponding to the 1 h hs-cTnI changes was 0.78, and when combining the 1 h hs-cTnI changes with the baseline value of the hs-cTnI upon presentation, the AUC further increased to 0.80. The authors concluded that the use of copeptin failed to provide any incremental diagnostic benefit to patients with troponin values falling into the “grey zone”, probably due to its non-specific nature [107].

Another sub-analysis of the APACE study compared various rule-out strategies for AMI based on high-sensitivity troponin assays, which were either hs-cTnT or hs-cTnI assays. Strategies under consideration included the following [108]: DMS using copeptin in combination with high-sensitivity troponin; strategy based on high-sensitivity troponin values < LoD [109]; 0/1 h, 0/2 h and 0/3 h ESC algorithms [24]; United Kingdom’s National Institute for Health and Care Excellence (NICE) algorithm [110]; and 2 h accelerated diagnostic protocol (2 h-ADP) algorithm [111]. All algorithms using hs-cTnT, except for the DMS, demonstrated similar diagnostic performance in terms of sensitivities and NPVs, which were in the ranges of 99.5–100% and 99.8–100%, respectively. On the other hand, the DMS using copeptin together with hs-cTnT exhibited a lower sensitivity of 96.7% and an NPV of 98.7%. Similar results with high and comparable sensitivities and NPVs were reported for all algorithms using hs-cTnI, apart from the NICE algorithm and the DMS based on copeptin in combination with hs-cTnI, which yielded much lower sensitivity and NPV [108]. However, it should be mentioned that the comparisons with the DMS were performed without excluding high-risk patients, as suggested by previous studies [86,102]. Interestingly, this study provides valuable clinical implications regarding the safety of all rule-out protocols, given that the rate of major adverse cardiac events was low and comparable among patients discharged by applying any of the aforementioned strategies [108].

A systematic review and meta-analysis of 14 observational studies investigated the supplemental diagnostic value of copeptin when used in conjunction with various troponin assays in a total of 7998 patients with suspected AMI. Overall, compared with the use of troponin alone, the combination of the two biomarkers (copeptin plus troponin) resulted in significant improvement of both sensitivity (from 0.81 to 0.92) and NPV (from 0.96 to 0.98). However, the addition of copeptin reduced specificity, as well as diagnostic accuracy, as evidenced by a decrease in the AUC value. On the other hand, when the analysis was refined to only eight studies using hs-cTnT, sensitivity increased significantly from 0.86 (with the use of hs-cTnT alone) to 0.93 (with the DMS), but NPV was reduced from 0.97 to 0.94. The addition of copeptin to hs-cTnT resulted in a decrease in diagnostic performance, as indicated by a decline in the AUC from 0.90 to 0.83. Therefore, copeptin provided an incremental value on the diagnostic performance of hs-cTnT only in terms of higher sensitivity [20].

A recent study compared the diagnostic accuracy for NSTEMI between a DMS (copeptin and hs-cTnT or hs-cTnI) and a strategy using baseline very low high-sensitivity troponin levels alone (hs-cTnT < 5 ng/L or hs-cTnI < 4 ng/L). The diagnostic benefit of the combined use of copeptin and hs-cTnT, as measured by the respective AUC, was equal (0.91) to the AUC of the hs-cTnT alone. In contrast, copeptin failed to convey any significant diagnostic benefit when added to hs-cTnI because it resulted in an AUC of 0.85 compared with an AUC of 0.93 for hs-cTnI alone. Sensitivities and NPVs did not differ between the DMS and the strategy of using very low high-sensitivity troponin levels alone. In the group of early presenters with chest pain onset <3 h, the sensitivity of the DMS slightly increased in comparison to the use of high-sensitivity troponin alone, albeit without reaching any statistical significance [112].

Pedersen et al. investigated the diagnostic performance of copeptin measured in the prehospital setting in addition to hs-cTnT measured upon hospital arrival. Comparing the DMS with the 0/3 h algorithm, the DMS resulted in a decreased length of stay by approximately 1 h without having any different impact on patient safety, as evidenced by equal incidence of major adverse cardiac events at 30 days in both groups. With regard to diagnostic utility, the DMS showed a sensitivity of 98.8% and an NPV of 99.6%, whereas the 0/3 h algorithm had a decreased sensitivity of 87.6% and an NPV of 98.9%. In a post hoc analysis, further comparisons were made between the DMS and two other more recently proposed diagnostic algorithms [113], namely a 0 h rule-out protocol (which rules out AMI if hs-cTnT levels are <LoD upon presentation) [114] and the 0/1 h algorithm proposed by the 2020 ESC guidelines [24]. The DMS yielded almost the same NPV as the other two strategies, while the sensitivity of the DMS was slightly lower, thus rendering the other two algorithms marginally superior to the DMS, albeit with their applicability being restricted to patients with chest pain onset >3 h [113].

Table 3 summarizes the main features of the studies, which either yielded controversial results or concluded that DMS is inferior to high-sensitivity troponin protocols.

#### 5.2.3. Copeptin in Early Presenters

In patients presenting early after chest pain onset, it has been suggested that copeptin may offer additional diagnostic information, which would allow for an earlier diagnosis of MI by performing one single measurement and thus circumventing the troponin-blind period. In the study by Stallone et al., the benefit of measuring copeptin along with hs-cTnT was evident in the group of early presenters (<2 h from chest pain onset). In this patient subgroup, the addition of copeptin increased the NPV for AMI from 92.9% when using hs-cTnT alone to 96% when using the combination of hs-cTnT and copeptin, while the sensitivity increased from 74.5% to 91.2%, respectively. With regard to the NPV, the added benefit of combining both biomarkers was mainly confined to those patients presenting within 1 h from chest pain onset, whereas it was no longer apparent in patients presenting after 2 h from chest pain onset. Nevertheless, when examining diagnostic accuracy in terms of ROC curves, the additional use of copeptin on top of hs-cTnT did not seem to provide any incremental value because it failed to increase AUC [105].

Likewise, a subgroup of 105 early presenters (<2 h from chest pain onset) was further analyzed in a prospective multicenter study, which sought to investigate the clinical utility of serial copeptin measurements upon presentation and at 1 h in 1439 patients presenting to the ED with chest pain. In these early presenters, it was observed that the addition of copeptin increased the NPV of hs-cTnT at presentation from 88.8% when using hs-cTnT alone to 95.9% when using the combination of the two biomarkers at presentation. A second copeptin measurement at 1 h did not provide any incremental value in terms of ruling out AMI because it did not result in further improvement of the NPV. However, a second hs-cTnT measurement did, in fact, improve the NPV when compared with the NPV of hs-cTnT alone upon presentation, but not when compared to the NPV of the combined biomarker strategy at baseline, thus indicating the valuable contribution of copeptin in excluding AMI in the group of early presenters [106].

Additionally, a post hoc analysis of three prospective French studies was performed rather recently, which included 449 patients presenting to the ED with chest pain of less than 6 h in duration. The aim was to investigate the diagnostic performance of a strategy based on a single measurement of hs-cTnT and copeptin upon admission in early presenters with suspected NSTEMI. The cut-off value for copeptin was set at 12 pmol/L, whereas the performance of hs-cTnT was tested at three different thresholds, namely <14 ng/L (99th percentile), <5 ng/L (LoD) and <3 ng/L (LoB). Patients were classified into three groups according to the time from symptom onset: early presenters with chest pain onset <2 h, those with chest pain onset between 2 and 4 h, and those with chest pain onset >4 h. In the group of early presenters, the diagnostic accuracy of hs-cTnT alone was 0.853, but with the addition of copeptin, it increased to 0.897. Furthermore, when the authors used the cut-off value of <14 ng/L for hs-cTnT and compared the diagnostic performance of hs-cTnT alone vs. the combination of copeptin and hs-cTnT, it was observed that the sensitivity increased from 80% to 93% and the NPV from 98% to 99%, but the specificity decreased substantially (from 85% to 54%). Even when reducing the diagnostic threshold of hs-cTnT to <5 ng/L and <3 ng/L, the sensitivity of hs-cTnT alone increased from 80% to 87% in both cases, but the sensitivity of the DMS did not change in either case and remained at 93%, which is regarded as suboptimal for a safe rule-out of patients with suspected AMI. In the group of patients with chest pain onset between 2 and 4 h, when compared with hs-cTnT alone (using a cut-off of <14 ng/L), the combination of copeptin with hs-cTnT resulted in an increase of sensitivity from 77% to 95% and an increase of NPV from 95% to 99%. Notably, when using lower cut-offs for hs-cTnT (either LoD or LoB), the sensitivities and the NPVs of the combined biomarker strategy reached 100% for both hs-cTnT thresholds, albeit at the expense of specificity. Finally, in the group of patients arriving >4 h after chest pain onset, hs-cTnT outperformed DMS in terms of diagnostic performance. Another finding of the study was that, as time from symptom onset increased from <2 h to >4 h, the diagnostic performance of the stand-alone use of hs-cTnT also increased, whereas the diagnostic performance of the combined use of hs-cTnT and copeptin remained rather unchanged across all categories of chest pain onset. All in all, the DMS did not demonstrate sufficient diagnostic accuracy in terms of safely ruling out AMI in very early presenters, while it seemed to have potential use in patients presenting after 2 h from symptom onset. It should be noted that, although copeptin was measured on the Kryptor analyzer, which uses the newer TRACE-based technology, the sensitivity of the assay was suboptimal, with an LoD of 4.8 pmol/L (instead of the 1.9 pmol/L level recommended by the manufacturer) and a functional assay sensitivity (20% CV) of 12 pmol/L [104].

Table 4 summarizes evidence regarding the diagnostic value of copeptin in early presenters.

#### 5.2.4. Copeptin in Combination with Risk Stratification Scores

A core component in the diagnostic approach of patients with suspected AMI is the assessment of clinical characteristics. Clinical scores, such as GRACE [99] and HEART [98] scores, have been proposed as objective tools that can be used to quantify clinical, electrocardiographic and laboratory features for the risk stratification of patients with chest pain. Accordingly, the diagnostic utility of copeptin has been examined in conjunction with these clinical risk scores and has yielded promising results.

A prospective observational study, which included 247 patients with symptoms suggestive of AMI, assessed the incremental value of copeptin when combined with hs-cTnT and GRACE score upon ED presentation. Copeptin < 14 pmol/L, hs-cTnT < 14 ng/L and a GRACE score < 108 were used as diagnostic thresholds for the three variables. Among various combinations (hs-cTnT alone, copeptin combined with hs-cTnT, hs-cTnT combined with GRACE score, and copeptin combined with both hs-cTnT and GRACE score), the latter yielded the best diagnostic accuracy, as reflected by 98% sensitivity and 99% NPV [115].

In another study including a cohort of 154 patients with suspected ACS [116], the modified HEART score (mHS), which comprises age, ECG features, number of risk factors and related symptoms [98], was added to either baseline hs-cTnT values or to the DMS (hs-cTnT and copeptin). The aim was to investigate the diagnostic accuracy of each individual rule-out algorithm, namely hs-cTnT alone, hs-cTnT combined with mHS and hs-cTnT combined with copeptin and mHS. It was observed that the triple combination of hs-cTnT < 14 ng/L, copeptin < 17.4 pmol/L and mHS ≤ 3 outperformed the other two algorithms because it effectively and safely ruled out 77% of patients with a significantly higher sensitivity of 100% and an NPV of 100% [116].

Studies examining the diagnostic performance of copeptin combined with clinical risk stratification scores are summarized in Table 5.

## 6. Discussion

Copeptin has emerged as an adjunctive biomarker on top of troponin in an attempt to improve diagnostic accuracy during the diagnostic approach of patients presenting to the ED with chest pain suggestive of AMI. Currently, its incremental diagnostic value is beyond doubt when used in combination with conventional troponin. Nonetheless, its diagnostic value has been questioned with the advent of the fourth-generation troponin assays, which display enhanced sensitivity. Although the 2020 ESC guidelines for NSTEMI [24] have taken into account valuable scientific studies [93,95,97,105,106,107,108] in order to recommend against the use of copeptin in combination with high-sensitivity troponin assays, it seems that several supporting data in favor of the supplemental value of copeptin on top of high-sensitivity troponins, presented in the systematic review of Raskovalova et al. [89] and in other studies [86,93,97,100,102], have been underappreciated, as thoroughly outlined in a critical appraisal of the ESC guidelines [25].

Even with the use of refined high-sensitivity assays, clinical decision-making still remains cumbersome, given that no ideal diagnostic method exists that is void of downsides. Concerns about misdiagnosis using high-sensitivity troponin assays [117], as well as the implementation of fast algorithms for ruling in and ruling out an AMI, may be overwhelming for clinicians, especially when discharging a patient from the ED [118]. On these grounds, it has been aptly cited that “when troponin was a lousy assay, it was a great test, but now that it’s becoming a great assay, it’s getting to be a lousy test” [119], which essentially reflects the skepticism about the strict adherence to troponin values for the assessment of patients with chest pain.

In addition, there seem to be several shortcomings when applying accelerated protocols. First, a main prerequisite for applying such protocols is the duration of chest pain, which needs to be more than 3 h long. Bearing this in mind, it becomes evident that early presenters are automatically precluded from the application of an accelerated diagnostic protocol. Notably, early presenters constitute a considerable proportion of patients with chest pain, ranging from 21.8% to 36% based on reports from multiple observational studies [104,105,112,120]. Second, practical issues arise when considering the turnaround time for the result acquisition, especially with the application of the 0/1 h protocol. A blind draw of the second blood sample is recommended by the ESC taskforce in order to resolve such practical difficulties. However, a blind blood draw for a second troponin measurement at 1 h may incur unnecessary additional costs, which could have been otherwise avoided in approximately 10–15% of patients who could have been actually ruled out with only one troponin measurement below the level of detection [24]. Third, systematic implementation is erratic in institutions where high-sensitivity troponins are not available. According to real-world data from 2000 institutions across five continents, only 41% of health centers worldwide use high-sensitivity troponin assays [121]. This fact highlights the difficulties in broadly adopting fast diagnostic protocols. Last, but not least, by applying fast rule-out protocols, a considerable proportion of patients (22–25%) [122,123] fall into the “grey zone”, which practically translates to longer waiting times, delayed diagnosis and suboptimal management.

The rationale for following an expedited diagnostic approach in patients with symptoms suggestive of AMI lies in the early rule-out of patients with other causes of chest pain while simultaneously avoiding unnecessary testing and long waiting times in the ED. As a consequence, this could aid in preserving human and hospital resources for other critically ill patients. A fast diagnostic protocol is also warranted for the early rule-in of patients with NSTEMI so as to tailor appropriate therapy in a timely manner.

Numerous studies provide evidence of the incremental value of copeptin in combination with high-sensitivity troponin assays. Its superiority over established diagnostic algorithms [78,92,93,100,101,102,103] may be slight yet significant. In fact, the effectiveness of the DMS has been highlighted in a cost analysis study, which compared it to standard diagnostic work-up (serial troponin measurements). The study concluded that the strategy of single combined measurement of copeptin and troponin in patients with suspected ACS is not only safe but also saves healthcare costs and hospital resources, reduces the hospital staff’s workload, and decreases the length of stay and overcrowding in the ED. Furthermore, DMS implementation seems to decrease the proportion of patients allocated for inpatient medical care [124]. In the era of high-sensitivity troponin assays, the strongest argument in support of the inclusion of copeptin in a biomarker rule-out strategy is its ease of applicability, as, in contrast to troponin, it is not influenced by gender, age, renal function and time from symptom onset. Moreover, DMS-based decision-making requires only one single measurement of both biomarkers, thus obviating the need for a second blood draw. Accordingly, the DMS could substantially facilitate the decision-making process in certain patient groups because it could more accurately lead to a definite decision in patients presenting to the ED in <3 h from symptom onset, in patients classified into the grey-zone based on existing recommended algorithms, as well as in patients with comorbidities.

In addition, biomarkers may serve as important prognostic tools for the recognition and risk stratification of adverse periprocedural ischemic events, as recently demonstrated in an observational study that focused on the prognostic importance of high-sensitivity troponin levels [125]. Along the same lines, a retrospective study of 149 patients reported that high levels of copeptin after percutaneous coronary intervention were associated with adverse outcomes during long-term follow-up [126]. The significant prognostic value of copeptin in predicting short- and long-term mortality in patients with AMI has also been highlighted in a systematic review and meta-analysis [127]. Because copeptin is a marker of endogenous stress, it would be reasonable to further investigate not only its diagnostic value in the setting of AMI but also its prognostic utility during the periprocedural period.

In order to determine the net benefit of copeptin, future studies should focus on the diagnostic performance of the DMS as a rule-out strategy exclusively in low-to-intermediate risk patients in terms of sensitivity, NPV and, primarily, safety. In addition, further studies should address specific populations, such as elderly patients or patients with comorbidities (cardiovascular or chronic kidney disease), in order to explore whether copeptin could resolve diagnostic ambiguities arising from false-positive troponin values. Furthermore, standardization of technical and statistical issues, such as the type of the copeptin assay and the appropriate cut-off value, may aid in a better understanding of the copeptin’s contribution to the decision-making process. Nevertheless, the utility of a DMS, which incorporates measurement of copeptin in conjunction with troponin, seems promising and deserves further consideration. This necessity becomes even more plausible, especially when taking into account the fact that both the numeral and the diagnostic burden of patients with chest pain in the ED could possibly be overcome by the implementation of a fast yet safe protocol of combined biomarkers regardless of time from symptom onset and institutional difficulties.

## 7. Conclusions

Undoubtedly, copeptin has an established role in patients with suspected ACS in terms of providing added diagnostic value when used in combination with conventional troponin. However, its diagnostic utility, when combined with high-sensitivity troponin, is still a matter of ongoing debate. Even though high-sensitivity troponin assays have allowed for earlier identification of patients with AMI, optimization of biomarker strategies still remains an unmet need, especially in equivocal clinical scenarios and populations with suspected AMI. Therefore, further research is warranted in order to decipher the true range of copeptin’s added diagnostic value in the modern era of refined cardiac troponin assays.

## Figures and Tables

**Table 1:** Studies providing supporting evidence for the incremental diagnostic value of copeptin in combination with conventional troponin.

Study (Author, Year)	Type of Study	No. of Pts	Cut-Offs/Protocol	Rule-Out Population	SMS			DMS			Key Findings
					AUC	Sensitivity (%) (95% CI)	NPV (%) (95% CI)	AUC	Sensitivity (%) (95% CI)	NPV (%) (95% CI)	
Reichlin et al., 2009 [75]	Prospective single center	487	cnv cTnT < 0.01 μg/L ± Copeptin < 14 pmol/L	Overall	0.86 (0.80–0.92)			0.97 (0.95–0.98)	98.8	99.7	DMS superior to SMS
Keller et al., 2010 [85]	Multicenter	1386	cnv cTnT < 0.03 μg/L ± Copeptin < 9.8 pmol/L	Overall	0.84 (0.82–0.87)	62 (56.2–67.5)	88.5 (86.4–90.4)	0.93 (0.92–0.95)	90.9 (87.1–93.9)	95.8 (93.9–97.2)	DMS superior to SMS
				CPO < 3 h	0.77 (0.72–0.82)	43 (34–52.3)	82.4 (78.3–86)	0.9 (0.88–0.93)	85.1 (77.5–90.9)	92.4 (88.2–95.4)	DMS superior to SMS in the early presenters
				All pts except for STEMI pts	0.87	64.7	92.4	0.93	89.3	96.5	DMS superior to SMS also after excluding STEMI pts
				CPO < 3 h excluding STEMI pts	0.79	46.7	89.0	0.9	81.3	94.0	DMS superior to SMS also after excluding STEMI pts
Lipinski et al., 2014 [88]	Systematic review and meta-analysis	9244	hs-cTn ± copeptin		0.912 (0.87–0.95)	0.878 (0.85–0.89)	0.962 (0.95–0.96)	0.795 (0.64–0.94)	0.957 (0.943–0.969)	0.982 (0.97–0.98)	DMS superior to SMS in terms of sensitivity and NPV but not in the overall AUC Only the combination of copeptin with cnv cTn significantly improved diagnostic accuracy, as assessed by the pooled area under the ROC curve.
			cnv cTn ± copeptin		0.885 (0.855–0.916)	0.686 (0.661–0.710)	0.930 (0.924–0.936)	0.856 (0.789–0.924)	0.905 (0.888–0.921)	0.970 (0.964–0.975)	
Raskovalova et al., 2014 [89]	Systematic review and meta-analysis	8740	cTn ± copeptin	Overall	-	0.87 (0.80–0.95)	-	-	0.96 (0.93–0.99)	-	DMS superior to SMS in terms of sensitivity, regardless of the troponin assay used
		6534	cnv cTnT ± copeptin	After exclusion of STEMI pts	-	0.80 (0.73–0.88)	-	-	0.95 (0.91–0.98)	-	
		4330	hs-cTnT ± copeptin		-	0.91 (0.86–0.97)	-	-	0.98 (0.96–1.00)	-	
		3996	cTnI ultra ± copeptin		-	0.79 (0.74–0.85)	-	-	0.93 (0.88–0.98)	-	
Balmelli et al., 2013 [87]	Prospective multicenter	1247	cnv cTnT < 0.035 μg/L hs-cTnT < 14 ng/L ± Copeptin <18.9 pmol/L	Overall	cnv cTnT 0.90 (0.84–0.95)			0.96 (0.94–0.98)			DMS equal to SMS for predicting 1 y mortality No gender differences in AUC when comparing SMS vs. DMS
					hs-cTnT 0.94 (0.91–0.98)			0.96 (0.93–0.98)			
				After exclusion of STEMI pts	cnv cTnT 0.83 (0.77–0.90)			0.89 (0.83–0.95)			
					hs-cTnT 0.92 (0.88–0.96)			0.92 (0.88–0.96)			
Maisel et al., 2013 [19]	Prospective multicenter	1967	cnv cTnI <40 ng/L ± Copeptin <14 pmol/L	CPO < 6 h	0.86			0.97	92.2 (85.9–95.9)	99.2 (98.5–99.6)	DMS allows safe rule-out of AMI with an NPV > 99% DMS provided prognostic information, with copeptin being a stronger predictor of short-term (<30 d) mortality and cnv cTnI of long-term (>60 d) mortality
Möckel et al., 2015 [86]	Multicenter RCT	902	POC cTnT < 30 ng/L hs-TnT < 14 ng/L cnv cTnI < 56 ng/L cnv cTnI < 45 ng/L ± copeptin < 10 pmol/L	Overall, SMS (serial cTn measurements) vs. DMS (cTn plus copeptin)	-	-	-	-	-	-	DMS equally safe to SMS 30 d MACE did not differ between SMS and DMS (5.17% vs. 5.19%) ↓ median LOS with DMS, 4 h (2–6), vs. 7 h (4–9) with SMS DMS: ↑ safe ED discharge rate

Abbreviations: AUC = area under the curve; CI = confidence interval; cnv cTn = conventional cardiac troponin; cnv cTnI = conventional troponin I; cnv cTnT = conventional troponin T; CPO = chest pain onset; cTn = cardiac troponin; d = day(s); DMS = dual marker strategy; ED = emergency department; h = hour(s); hs-cTn = high-sensitivity cardiac troponin; hs-cTnT = high-sensitivity cardiac troponin T; LOS = length of stay; MACEs = major adverse cardiovascular events; NPV = negative predictive value; POC = point of care; pts = patients; RCT = randomized controlled trial; ROC = receiver operating characteristic; SMS = single marker strategy; STEMI = ST segment elevation myocardial infarction; vs. = versus; y = year; ↓: decrease; ↑: increase.

**Table 2:** Studies suggesting that dual marker strategy is superior or equal to high-sensitivity troponin protocols.

Study (Author, Year)	Type of Study	No. of Pts	Cut-Offs/Protocol	Rule-Out Population/Protocol	SMS			DMS			Key Findings
					AUC	Sensitivity (%) (95% CI)	NPV (%) (95% CI)	AUC	Sensitivity (%) (95% CI)	NPV (%) (95% CI)	
Zellweger et al., 2015 [93]	Prospective multicenter	379	hs-cTnT < 14 ng/L ± Copeptin < 9 pmol/L	Diabetic pts	0.90 (0.86–0.93)	-	-	0.90 (0.87–0.93)	-	-	Diagnostic accuracy equal for SMS and DMS DMS improved risk stratification and prediction of 2y mortality
Mueller-Hennessen et al., 2019 [97]	Prospective international multicenter	922	hs-cTnT < 14 ng/L ± Copeptin <10, <14, or <20 pmol/L		0.92 (0.90–0.94)	89.0 (82.9–93.4)	97.4 (95.9–98.5)	0.93 (0.91–0.95)	93.5–94.8	98.1–98.3	DMS showed comparable diagnostic performance with the other rule-out algorithms DMS resulted in a rapid and safe rule-out in up to 62.3% of pts The addition of HEART/GRACE score to DMS led to improved NPV and sensitivity
			LoB (hs-cTnT < 3 ng/L)			98.7 (95.4–99.8)	99.4 (97.8–99.9)				
			LoD (hs-cTnT < 5 ng/L)			98.1 (94.4–99.6)	99.3 (97.9–99.9)				
			1 h algorithm			96.8 (92.6–98.9)	99.2 (98.1–99.7)				
			ESC algorithm			98.7 (95.4–99.8)	99.5 (98.0–99.9)				
Sebbane et al., 2013 [78]	Prospective single center	194	hs-cTnT >14 ng/L ± us copeptin > 13.11 pmol/L	Overall CPO < 12 h	0.89 (0.85–0.92)	76.9 (63.2–87.5)	91 (84.8–95.3)	0.93 (0.89–0.97)	96.2 (86.8–99.5)	97.8 (92.4–99.7)	DMS superior to SMS
				After excluding STEMI pts		76 (54.9–90.6)	95.3 (90–98.2)		96 (79.6–99.9)	98.9 (94–100)	
Potocki et al., 2012 [92]	Prospective multicenter	1170	cnv-cTnT 0.01 ng/L hs-cTnT < 14 ng/L ± Copeptin < 9 pmol/L	Pre-existing CAD (433 pts)	0.92 (0.89–0.96)	93.6 (85.7–97.9)	97.7 (94.8–99.3)	0.94 (0.91–0.97)	98.7 (93.0–99.8)	99.3 (96.3–99.9)	DMS (hs-cTnT + copeptin) showed a trend to superiority compared with hs-cTnT alone Copeptin was an independent predictor of 1 y mortality
				No CAD (737 pts)	0.96	94.3 (88.1–97.9)	98.9 (97.5–99.6)	0.97	99.1 (94.8–99.8)	99.7 (98.5–100.0)	
Stengaard et al., 2017 [94]	Retrospective	962	hs-cTnT < 14 ng/L ± Copeptin < 9.8 pmol/L Prehospital setting	Overall	0.81 (0.78–0.85)	80 (73–85)	93 (91–96)	0.85 (0.83–0.88)	96 (91–98)	98 (96–99)	DMS superior to SMS DMS application is feasible in the prehospital setting and may improve pts’ prehospital triage
				CPO < 1 h	0.75 (0.69–0.82)	67 (55–78)	91 (87–94)	0.84 (0.79–0.88)	97 (90–100)	99 (95–100)	
Giannitsis et al., 2019 [102]	Prospective multicenter	2294	cnv cTn or hs-cTn < 99^th^ percentile ± Copeptin < 10 pmol/l							99.9 (NPV for excluding NSTEMI)	30 d mortality was lower in the DMS group compared with the SMS group (0.1% vs. 1.1%) DMS group had lower LOS than the SMS group (228 vs. 288 min)
Chenevier-Gobeaux et al., 2019 [104]	post hoc analysis	449	hs-cTnT < 3 ng/L, or <5 ng/L, or <14 ng/L ± Copeptin < 12 pmol/L	hs-cTnT < 14 ng/L CPO < 2 h	0.853 (0.789–0.904)	80 (51–95)	98 (93–100)	0.897 (0.84–0.94)	93 (66–100)	99 (93–100)	DMS equal to SMS, but neither safe enough in very early presenters
				hs-cTnT < 14 ng/L CPO 2–4 h	0.869 (0.802–0.919)	77 (54–91)	95 (88–98)	0.891 (0.829–0.937)	95 (75–100)	99 (91–100)	
Giannitsis et al., 2020 [101]	Retrospective	10,329	hs-cTnT < 5 ng/L or <14 ng/L hs-cTnI < 2 ng/L or <34 ng/L ± Copeptin < 10 pmol/L or <14 pmol/L	hs-cTnT < 14 ng/L CPO 2–4 h		77 (54–91)	95 (88–98)		95 (75–100)	99 (91–100)	DMS similar to SMS (sensitivity/NPV) Eligibility for rule-out was 2.4 fold higher with DMS than SMS (61.4% vs. 25.3%)
Kim et al., 2020 [96]	Prospective single center	263	hs-cTnI > 26.2 ng/L ± Copeptin > 10 pmol/L	0 h	0.914 (0.873–0.955)	96.4 (81.7–99.9)	99.5 (97.3–100)	0.840 (0.811–0.870)	100 (87.7–100)	100 (97.7–100)	DMS equal to SMS for NSTEMI diagnosis
				2 h	0.928 (0.905–0.950)	100 (87.7–100)	100 (98.2–100)				
				0/2 h	0.928 (0.905–0.950)	100 (87.7–100)	100 (98.2–100)				
Ricci et al., 2022 [103]	Prospective single center	1136	hs-cTnI ≤ 27 ng/L ± Copeptin < 10 pmol/L + low risk ECG OR hs-cTnI < 5 ng/L + low risk ECG	DMS 0-h		97.8% (95–99.3)	98.7 (96.9–99.6)		95.2 (91.5–97.6)	97.4 (95.4–98.7)	DMS equal to SMS and ESC 0/1 h algorithm in terms of safety Discharge rate: 37.4% with DMS, vs. 32.9% with SMS, vs. 35.4% with ESC 0/1 h

Abbreviations: AUC = area under the curve; CAD = coronary artery disease; CI = confidence interval; cnv cTnT = conventional troponin T; CPO = chest pain onset; cTn = cardiac troponin; d = day; DMS = dual marker strategy; ECG = electrocardiogram; ESC = European Society of Cardiology; h = hour; hs-cTnI = high-sensitivity cardiac troponin I; hs-cTnT = high-sensitivity cardiac troponin T; LoB = limit of blank; LoD = limit of detection; LOS = length of stay; min = minutes; NPV = negative predictive value; NSTEMI = non-ST segment elevation myocardial infarction; pts = patients; SMS = single marker strategy; us copeptin = ultra-sensitive copeptin; vs. = versus; y = year(s).

**Table 3:** Studies yielding controversial results or showing that DMS is inferior to high-sensitivity troponin protocols.

Study (Author, Year)	Type of Study	No. of Pts	Cut-Offs/Protocol	Rule-Out Population/Protocol	SMS			DMS			Key Findings
					AUC	Sensitivity (%) (95% CI)	NPV (%) (95% CI)	AUC	Sensitivity (%) (95% CI)	NPV (%) (95% CI)	
Stallone et al., 2016 [105]	Prospective multicenter	2000	hs-cTnT < 14 ng/L ± Copeptin < 9 pmol/L	CPO < 2 h	0.87 (0.83–0.90)	75 (65–83)	93 (90–95)	0.86 (0.82–0.90)	91 (84–96)	96 (93–98)	AUC difference not significant
				CPO > 2 h	-	96 (92–98)	99 (98–99)	-	99 (97–100)	99 (99–100)	
Hillinger et al., 2015 [106]	Prospective multicenter	1.439	hs-cTnT < 14 ng/L ± Copeptin < 10 pmol/L	0 h biomarkers	-	-	97.1 (95.9–98.1)	-	-	98.6 (97.4–99.3)	Additive value of 1 h copeptin—not significant 1 h hs-cTn improves AMI diagnosis
				1 h biomarkers	-	-	99.6 (98.7–99.9)	-	-	98.6 (97.3–99.3)	
				CPO < 2 h			88.8 (80.3–94.5)			95.9 (86.0–99.5)	
Boeddinghaus et al., 2017 [107]	Prospective single center	1356	hs-cTnI < 26.2 ng/L hs-cTnT < 14 ng/L ± Copeptin < 9.8 pmol/L	0 h hs-cTnI	0.51 (0.39–0.64)	-	-	0.52 (0.39–0.65)	-	-	No incremental diagnostic benefit conferred by copeptin 1 h hs-cTnI changes plus 0 h hs-cTnI improved AUC to 0.80
				1 h hs-cTnI	0.78 (0.68–0.88)						
Wildi et al., 2019 [108]	Prospective international multicenter	3696	hs-cTnI/T < 99th percentile ± Copeptin < 9 pmol/L	hs-cTnT	-	99.5–100	99.8–100	-	96.7 (94.2–98.1)	98.7 (97.8–99.3)	DMS was inferior to cTn strategies
				hs-cTnI	-	98.9–100	99.7–100	-	90.4 (86.8–93.3)	96.9 (95.6–97.8)	
Shin et al., 2018 [20]	Systematic review and meta-analysis	7.998	cnv cTn or hs-cTn ± Copeptin		0.91 (0.90–0.91)	0.81 (0.74–0.87)	0.96 (0.95–0.98)	0.85 (0.83–0.86)	0.92 (0.89–0.95)	0.98 (0.96–0.99)	DMS superior to SMS (sensitivity, NPV) yet inferior regarding overall diagnostic accuracy (AUC)
				hs-cTnT ± copeptin	0.90 (0.88–0.92)	0.86 (0.79–0.93)	0.97 (0.95–0.99)	0.83 (0.80–0.86)	0.93 (0.91–0.96)	0.94 (0.89–0.98)	
Restan et al., 2022 [112]	Two cohorts	959	hs-cTnT < 5 ng/L or hs-cTnI < 4 ng/L ± Copeptin < 9 pmol/L	Overall hs-cTnT hs-cTnI	0.91 (0.89–0.93) 0.93 (0.91–0.95)	98.9 (94.0–100) 97.8 (92.2–99.7)	99.6 (97.0–99.9) 99.5 (97.9–99.9)	0.91 (0.89–0.93) 0.85 (0.82-.87)	98.9 (94.0–100) 97.8 (92.2–99.7) (*p* = 1.0)	99.5 (96.7–99.9) 99.4 (97.5–99.8) (*p* < 0.001)	DMS not superior to ESC 0/1 algorithm
				CPO <3 h hs-cTnT hs-cTnI	0.838 (0.78–0.88) 0.890 (0.84–0.93)	97.1 (85.1–99.9) 91.4 (76.9–98.2)	98.5 (90.3–99.8) 97.1 (91.7–99.0)	0.846 (0.79–0.89) 0.901 (0.85–0.93)	100 (90.0–100) (*p* = 0.32) 100 (90.0–100) (*p* = 0.08)	100 (*p* = 0.38) 100 (*p* = 0.13)	AUC difference not significant Sensitivity improvement not significant
Pedersen et al., 2023 [113]	Multicenter RCT	4351	Prehospital Copeptin < 9.8 pmol/L ± hs-cTnT < 14 ng/L	Overall, 0/3 h algorithm		87.6 (81.3–92.4)	98.9 (98.3–99.3)		98.8 (94.2–99.4)	99.6 (99.0–99.9)	DMS marginally inferior to 0/1 h algorithm and 0 h LoD rule-out protocol DMS ↓ mean LOS by 0.9 h (95% CI 0.7–1.1 h) DMS non-inferior to SMS for 30 d MACE
		1585 subgroup		0/1 h		99.2 (95.9–100.0)	99.9 (99.4–100.0)		98.5 (94.6–99.8)	99.7 (99.0–100.0)	
				0 h hs-cTnT <LoD		99.2 (95.9–100.0)	99.6 (97.7–100.0)				

Abbreviations: AMI = acute myocardial infarction; AUC = area under the curve; CI = confidence interval; cnv cTn = conventional cardiac troponin; CPO = chest pain onset; cTn = cardiac troponin; d = day; DMS = dual marker strategy; h = hour; hs-cTn = high-sensitivity cardiac troponin; hs-cTnI = high-sensitivity cardiac troponin I; hs-cTnT = high-sensitivity cardiac troponin T; LoD = limit of detection; LOS = length of stay; min = minutes; MACEs = major adverse cardiac events; NPV = negative predictive value; NSTEMI = non-ST segment elevation myocardial infarction; pts = patients; RCT = randomized controlled trial; SMS = single marker strategy; vs. = versus; ↓ decrease.

**Table 4:** Studies providing evidence on the diagnostic utility of copeptin in early presenters.

Study (Author, Year)	Type of Study	Number of Patients	Cut-Offs	Rule-Out Protocol	SMS			DMS			Key Findings
					AUC	Sensitivity (%) (95% CI)	NPV (%) (95% CI)	AUC	Sensitivity (%) (95% CI)	NPV (%) (95% CI)	
Stallone et al., 2016 [105]	Prospective multicenter	519	hs-cTnT < 14 ng/L ± Copeptin < 9 pmol/L	CPO < 2 h	0.87 (0.83–0.90)	74.5 (64.9–82.6)	92.9 (89.8–95.3)	0.86 (0.82–0.90)	91.2 (84–95.9)	96 (92.5–98.2)	DMS increased sensitivity and NPV but failed to improve diagnostic accuracy in terms of AUC
Hillinger et al., 2015 [106]	Prospective multicenter	105	hs-cTnT < 14 ng/L ± Copeptin < 10 pmol/L	CPO < 2 h			88.8 (80.3–94.5)			95.9 (86–99.5)	DMS better than SMS for rule-out upon presentation (0 h) DMS showed equal diagnostic value to 0/1 h hs-cTnT protocol No additional benefit of second measurement of copeptin at 1 h
Stengaard et al., 2017 [94]	Retrospective	395	hs-cTnT < 14 ng/L ± Copeptin < 9.8 pmol/L	CPO < 1 h Prehospital setting	0.75 (0.69–0.82)	67 (55–78)	91 (87–94)	0.84 (0.79–0.88)	97 (90–100)	99 (95–100)	DMS superior to SMS for prehospital rule-out of very early presenters
Chenevier-Gobeaux et al., 2019 [104]	Post hoc analysis	303 160 pts with CPO < 2 h 143 pts with CPO between 2–4 h	hs-cTnT < 3 ng/L, or <5 ng/L, or <14 ng/L ± Copeptin < 12 pmol/L	CPO < 2 h; hs-cTnT < 14 ng/L	0.853 (0.789–0.904)	80 (51–95)	98 (93–100)	0.897 (0.84–0.94)	93 (66–100)	99 (93–100)	DMS increased sensitivity and reduced the number of misclassified pts compared with SMS No strategy was safe enough for ruling out NSTEMI in very early presenters
				CPO 2–4 h; hs-cTnT < 14 ng/L	0.869 (0.802–0.919)	77 (54–91)	95 (88–98)	0.891 (0.829–0.937)	95 (75–100)	99 (91–100)	

Abbreviations: AUC = area under the curve; CI = confidence interval; CPO = chest pain onset; DMS = dual marker strategy; h = hour(s); hs-cTnT = high-sensitivity cardiac troponin T; NPV = negative predictive value; NSTEMI = non-ST segment elevation myocardial infarction; pts = patients; SMS = single marker strategy.

**Table 5:** Studies providing evidence on the diagnostic utility of copeptin in combination with clinical risk stratification scores.

Study (Author, Year)	Type of Study	Number of Patients	Cut-Offs	Rule-Out Protocol	Baseline hs-cTnT		Combined Strategies		Key Findings
					Sensitivity (%) (95% CI)	NPV (%) (95% CI)	Sensitivity (%) (95% CI)	NPV (%) (95% CI)	
Bohyn et al., 2014 [115]	Prospective observational	247	hs-cTnT < 14 ng/L ± Copeptin < 14 pmol/L	SMS (baseline hs-cTnT) vs. DMS (hs-cTnT + copeptin)	72 (58–83)	92 (88–95)	90 (79–96)	95 (90–98)	DMS superior to SMS The addition of GRACE score further improves diagnostic accuracy
				DMS + GRACE score < 108			98 (90–100)	99 (94–100)	
Morawiec et al., 2018 [116]	Prospective	154	hs-cTnT < 14 ng/L ± Copeptin < 17.4 pmol/L	Baseline hs-cTnT alone vs. hs-cTnT + mHS ≤ 3	99.3 (88–97.9)	85.4 (70.8–94.4)	99.1 (94.8–100)	94.4 (72.2–99.9)	The triple combined strategy (hs-cTnT/copeptin/mHS) had the highest sensitivity and NPV for ruling out AMI It also had the best prognostic accuracy, providing optimal short-term risk stratification
				hs-cTnT + mHS ≤ 3 + copeptin			100 (96.6–100)	100 (75.3–100)	

Abbreviations: AMI = acute myocardial infarction; CI = confidence interval; DMS = dual marker strategy; hs-cTnT = high-sensitivity cardiac troponin T; mHS = modified HEART score; NPV = negative predictive value; SMS = single marker strategy.

## Data Availability

No new data were created or analyzed in this study.
